# Assessment of uncertainty quantification in universal differential equations

**DOI:** 10.1098/rsta.2024.0444

**Published:** 2025-04-02

**Authors:** Nina Schmid, David Fernandes del Pozo, Willem Waegeman, Jan Hasenauer

**Affiliations:** ^1^ Life & Medical Sciences (LIMES) Institute, University of Bonn, Bonn, Germany; ^2^ Department of Data Analysis and Mathematical Modelling, Ghent University, Ghent, Belgium; ^3^ Helmholtz Center Munich, German Research Center for Environmental Health, Computational Health Center, Munich, Germany

**Keywords:** uncertainty quantification, universal differential equations, scientific machine learning

## Abstract

Scientific machine learning is a new class of approaches that integrate physical knowledge and mechanistic models with data-driven techniques to uncover the governing equations of complex processes. Among the available approaches, universal differential equations (UDEs) combine prior knowledge in the form of mechanistic formulations with universal function approximators, such as neural networks. Integral to the efficacy of UDEs is the joint estimation of parameters for both the mechanistic formulations and the universal function approximators using empirical data. However, the robustness and applicability of these resultant models hinge upon the rigorous quantification of uncertainties associated with their parameters and predictive capabilities. In this work, we provide a formalization of uncertainty quantification (UQ) for UDEs and investigate key frequentist and Bayesian methods. By analyzing three synthetic examples of varying complexity, we evaluate the validity and efficiency of ensembles, variational inference and Markov-chain Monte Carlo sampling as epistemic UQ methods for UDEs.

This article is part of the theme issue ‘Uncertainty quantification for healthcare and biological systems (Part 2)’.

## 1. Introduction

Two primary paradigms govern the modelling of dynamical systems: (i) Mechanistic modelling, which builds on first principles to derive model structures and informs parameters using data; and (ii) machine learning (ML)-based modelling, which constructs governing equations directly from data. Prime examples of ML-based modelling of dynamical systems include composite artificial neural networks (ANNs) based on simple integrator schemes [1] and Runge–Kutta neural networks (RKNNs) [2]. This groundbreaking work led to the development of general neural ordinary differential equations (NODEs) [3]. Yet, while NODEs allow for uncovering governing equations of dynamical systems, they do not easily facilitate the integration of prior knowledge.

Scientific machine learning (SciML) unites the paradigms of mechanistic and ML-based modelling [4]. For instance, physics-informed neural networks (PINNs) describe solutions of dynamical systems using customized loss functions that favour predictions in agreement with first-principles mechanistic models [5]. Universal differential equations (UDEs) model the time derivative of the state of a system using a combination of terms derived from first principles and flexible ANNs [6]. In our opinion, UDEs stand out because they allow for the integration of prior knowledge and hard physical constraints (e.g. mass conservation or boundedness) [7], thus facilitating generalization. Moreover, they provide governing equations that can be further interrogated.

The parameters of both the mechanistic and the neural network components of a UDE are jointly estimated using data. Interpretation of modelling results hinges on quantifying uncertainties, encompassing mechanistic parameter values and predictions for the entire model or its components. Uncertainty quantification (UQ) of parameters is crucial because it provides insights into the reliability and range of potential values, allowing researchers to understand the robustness and credibility of their model’s mechanistic foundations. UQ of the prediction is equally important, as it offers a measure of the model’s reliability, e.g. for perturbation studies or scenario analysis, aiding decision-making by acknowledging the inherent uncertainty in forecasting outcomes.

UQ is a highly researched topic for both dynamical mechanistic modelling [8] and machine learning [9,10]. First-order uncertainty describes the inherent and irreducible stochasticity of the predictions (aleatoric uncertainty), while second-order uncertainty describes uncertainty originating from the uncertainty of parameter estimates (which is one component of epistemic uncertainty—see §2 (c)). By choosing a suitable noise model, the assessment of aleatoric uncertainty is well-defined. Hence, in this work, while reporting results on aleatoric uncertainty, we focus on estimating epistemic uncertainty.

In supervised machine learning, various methods aim to quantify epistemic uncertainty, and many of these methods have a resemblance to the field of mechanistic modelling. A fully Bayesian perspective is realized by Markov-Chain Monte Carlo (MCMC) sampling methods [11] and approximation methods like Variational Inference [12], yielding parameter distributions instead of point estimates. Deep ensembles [13] and multi-start ensembles in dynamic modelling [8,14] are both randomization-based ensemble approaches. Key differences between mechanistic modelling and machine learning are the number and interpretability of the parameters. Hence, some flavours of UQ methods are exclusively used in deep learning, like dropout as a Bayesian approximation [15]. Others are more common in dynamic modelling, like Profile Likelihood (PL) calculation [16] or asymptotic confidence intervals via the Fisher Information Matrix (FIM) [17].

For some modelling approaches in the field of SciML, like PINNs [18], a thorough investigation of UQ exists [19]. In contrast to other methods, UDEs embed neural networks directly in the differential equations. While this allows the incorporation of arbitrary levels of prior knowledge, it also yields challenges like over-parametrized differential equations with correlated parameters in combination with numerically more challenging simulations. To the best of our knowledge, previous work explored only basic UQ implementations for multi-start-optimization [20] or Bayesian neural networks [21,22] and only considered fully observed or densely measured state variables.

In this paper, we present several key contributions. Firstly, we introduce a formal definition of uncertainty tailored to UDEs, aiming to enhance precision and applicability in uncertainty assessments within this framework (§2). Secondly, we conduct an in-depth discussion of current epistemic UQ methods applicable to UDEs (§3). Lastly, we evaluate and compare the performance of a diverse set of UQ methods by investigating three synthetic examples (§4). Each synthetic example is implemented using several noise models, yielding eleven data scenarios in total. Synthetically generated data allows us to compare the methods’ results with an underlying ground truth. Our investigation spans considerations of computing time, estimations of aleatoric and epistemic uncertainty, parameter and prediction uncertainty, and different noise models, encompassing continuous and discrete distributions. Although the groups of UQ methods have been investigated before [8,23], we find novel insights in the context of UDEs.

## 2. Formalizing precision: a tailored definition of uncertainty for UDEs

In the following subsections, we first define the general setup of dynamic models, formally introduce UDEs and conclude by presenting different sources of uncertainty and discussing their relevance for UDEs.

### (a) Dynamic models

Let $x(t)\in {\mathbb{R}}^{n_{x}}$ be a time-dependent variable, denoting the state of a system at time t , that can be represented using a dynamic model. Dynamic models describe the value of x by parameterizing the derivative of x(t) and its initial condition $x(t_{0})$ using a vector field $f:\mathbb{R}\times {\mathbb{R}}^{n_{x}}\times {\mathbb{R}}^{n_{\theta }}\to {\mathbb{R}}^{n_{x}}$ :


(2.1) $$\begin{array}{lrr} & \begin{array}{rl} \frac{dx}{dt} & =f(t,x,\theta_{f}),\;x(t_{0})=x_{0}, \end{array} & \end{array}$$

where $\theta_{f}\in {\mathbb{R}}^{n_{\theta }}$ and $x_{0}\in {\mathbb{R}}^{n_{x}}$ are model parameters and initial conditions, respectively. Often, f is unknown and an estimate $\hat{f}$ is used instead. Let ${\hat{\theta }}_{f}$ and ${\hat{x}}_{0}$ be the parameters and initial conditions of $\hat{f}$ which we estimate based on $n_{t}$ discrete measurements at time points $\left\{t_{1},t_{2},...,t_{n_{t}}\right\}$ .

In many real-life scenarios, the state variables cannot be measured directly. Accordingly, the prediction of the differential equation model needs to be transformed using an observable function h to values predicting the measurable observables as:


(2.2) $$\begin{array}{lrr} & \begin{array}{rl} \hat{y}(t) & =h(\hat{x}(t))\in {\mathbb{R}}^{n_{y}}\;\text{with}\;\hat{x}(t)=\int_{t_{0}}^{t}\hat{f}(s,\hat{x}(s),{\hat{\theta }}_{f})\;ds+{\hat{x}}_{0}. \end{array} & \end{array}$$

Here, $\hat{x}(t)$ is the estimate of x(t) that we get from using $\hat{f}$ , ${\hat{\theta }}_{f}$ and ${\hat{x}}_{0}$ . An example for h comes from infectious disease modelling, where we often observe infections, but, e.g. not the number of susceptible, exposed or recovered persons.

Furthermore, measurements are subject to noise. Instead of measuring the underlying true value $\bar{y}(t_{k})=h(x(t_{k}))$ of the observable, we observe the random variable $y(t_{k})∼P$ with P being a probability distribution. In general, we do not know the underlying distribution of $y(t_{k})$ . Instead, we fit a parametric distribution, which is called the noise model. There exist different formulations for noise models, with the Gaussian being the most prominent representative. Depending on the characteristics of the underlying measurements like discreteness, overdispersion or skewness, other noise formulations may be more suitable. In the present work, we will focus on two commonly used noise models in the context of infectious disease modelling [24,25], the Gaussian noise model for continuous data and the Negative Binomial noise model for overdispersed and discrete data:

— **Gaussian noise model**: Let $\varepsilon (t_{k})∼N(0,\sigma^{2}I)$ . Then, we observe $y(t_{k})=\bar{y}(t_{k})+\epsilon (t_{k})$ , where σ is the constant standard deviation of the Gaussian distribution.
— **Negative Binomial noise model**: Let $y\in {\mathbb{R}}^{n_{y}}$ . The observed variable $y_{i}$ follows a Negative Binomial distribution with mean ${\bar{y}}_{i}(t_{k})$ and dispersion parameter d , i.e. $y_{i}(t_{k})∼\mathrm{NegBin}({\bar{y}}_{i}(t_{k}),d)$ , for all $i\in \left\{1,...,n_{y}\right\}$ .

In both cases, we assume that the i.i.d. assumption holds. Let $\theta =\left\{\theta_{f},\theta_{\text{np}}\right\}$ , where $\theta_{\text{np}}$ is the noise parameter of the respective noise model and p(y(t)|θ) the probability density function with mean value $\hat{y}(t)$ . Then, the objective of the optimization process is to maximize the likelihood of observing the data $D=\left\{(t_{i},y(t_{i})|i=1,...,n_{t}\right\}$ given the parameters θ .

### (b) Universal differential equations

UDEs combine known mechanistic terms $f_{\text{mech}}$ with universal function approximators (in this work neural networks) $f_{\text{net}}$ to describe the right-hand side of equation (2.1) [6]. For instance, the neural network can be used to describe the time-varying input of an otherwise purely mechanistic ordinary differential equation, i.e. for a fixed t we have $\hat{f}(t,x,\theta )={\hat{f}}_{\text{mech}}(t,x,\theta_{f})$ , with $\;\theta_{f}=\left(\theta_{\text{mech}},{\hat{f}}_{\text{net}}(t,\theta_{\text{net}})\right)$ . Alternatively, it can describe individual terms of the state derivatives, e.g. $\hat{f}(t,x,\theta )={\hat{f}}_{\text{mech}}(t,x,\theta_{\text{mech}})+{\hat{f}}_{\text{net}}(t,x,\theta_{\text{net}})$ . Hence, the formulation of UDEs allows us to incorporate arbitrary levels of mechanistic knowledge. Here, $\theta_{\text{net}}$ are the weights and biases of the neural network and $\theta_{\text{mech}}$ are the interpretable parameters of the mechanistic equation. Considering all parameters, we define $\theta =\left(\theta_{\text{mech}},\theta_{\text{net}},\theta_{\text{np}}\right)$ for scenarios in which the initial condition $x_{0}$ is known. The parameters are jointly estimated from data.

### (c) Sources of uncertainty

In general, we can (at least) formally identify two distinct types of uncertainty: *aleatoric* and *epistemic* uncertainty [9]. As they can guide the evaluation of model performance and its potential application to real-life scenarios, precise quantification of these types of uncertainty is essential. The aleatoric (statistical) uncertainty Var(y(t)) is based on inherent random effects and, hence, irreducible. By introducing a noise model, we aim to describe the aleatoric uncertainty. Epistemic (systematic) uncertainty stems from a lack of knowledge and potential model misspecifications. The bias-variance decomposition of the mean squared prediction error illustrates these different types of uncertainties [26]:


(2.3) $$\begin{array}{r} \begin{array}{rl} E_{y(t)}[E_{D}[(y(t)-\hat{y}(t){)}^{2}]] & =(E_{D}[\hat{y}(t)]-\bar{y}(t){)}^{2}+{Var}_{D}(\hat{y}(t))+Var(y(t))\\ & ={bias}^{2}+{Var}_{D}(\hat{y}(t))+Var(y(t)). \end{array} \end{array}$$

Hence, epistemic uncertainty can be decomposed further into model bias $\left(E_{D}[\hat{y}(t)]-\bar{y}(t)\right)$ and variance ${\mathrm{Var}}_{D}(\hat{y}(t))$ . As described in the previous section, we generally do not know f and θ . Uncertainty in the estimates $\hat{\theta }$ (model estimation) and $\hat{f}$ (model form) are sources of epistemic uncertainty. There exist various methods for the estimation of epistemic uncertainty, as discussed in §3. However, the model bias is often neglected by assuming $E_{D}[\hat{y}(t)]=\bar{y}(t)$ and reducing the epistemic uncertainty to approximation uncertainty. While we will follow this assumption, we cannot guarantee a negligible model uncertainty: SciML is typically applied to the low to medium data regime [27]. Although neural networks are universal approximators, making them asymptotically unbiased, a bias is typically still observed in the low to medium data regime [28,29].

UDEs are located at the interface of neural networks and mechanistic dynamical modelling. While regularization is vital for neural networks [30], so is the exhaustive exploration of the parameter space for mechanistic models where one is interested in a global solution. It is not trivial to find the right balance between these, which is one of the reasons why parameter uncertainty, i.e. estimation uncertainty, is of quite some importance for UDEs. Furthermore, the numerical precision of the ODE solver and data sparsity may influence the quality of parameter estimation.

## 3. Methodology for epistemic uncertainty quantification of UDEs

### (a) General setting

In this study, we will explore epistemic uncertainty arising as a result of parameter uncertainty, keeping the model form f fixed per problem setting. Bayes’ rule provides a formulation for this uncertainty. The posterior density p(θ|D) can be described in terms of the likelihood p(D|θ) and prior p(θ) :


(3.1) $$\begin{array}{r} p(\theta |D)=\frac{p(D|\theta )p(\theta )}{\int p(D|\theta )p(\theta )d\theta }. \end{array}$$

From this, the posterior predictive distribution is obtained as:


(3.2) $$\begin{array}{r} p(y(t)|D)=\int p(y(t)|\theta )p(\theta |D)d\theta . \end{array}$$

The prior distribution for the mechanistic parameters is usually chosen based on knowledge about the underlying processes. For the neural network parameters the choice of the prior distribution is less trivial. Commonly, an isotropic Gaussian prior is chosen [31]. Recently, it has been shown that especially for deep and flexible neural networks, this can cause drawbacks like the cold-posterior effect [32]. Specifying the correct prior is still a highly investigated research topic, and several options are discussed as alternatives for isotropic Gaussian priors [33]. One comparatively simple option is a Gaussian prior with a non-diagonal covariance matrix, allowing for correlation between different parameters [31].

### (b) Uncertainty quantification methods

**Figure 1.**
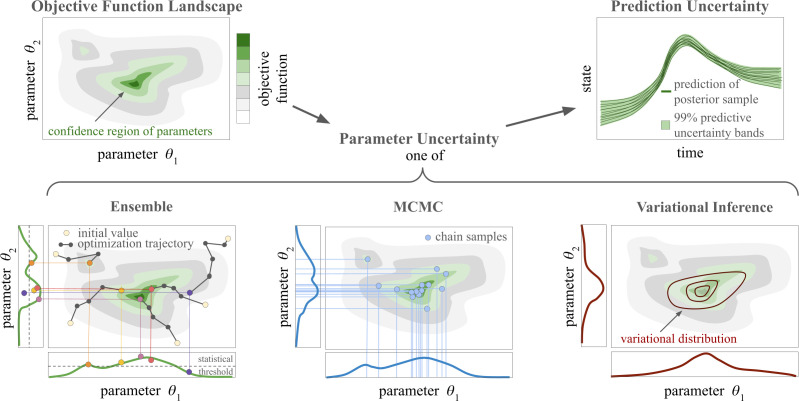
Overview of the presented uncertainty quantification methods. Given an unknown objective function landscape, we estimate the posterior distribution of the parameters using UDE ensembles, MCMC methods or Variational Inference. Based on random draws from the posterior distribution, predictions of the state trajectory are made, yielding lower and upper bounds for a 99% prediction interval.

In the design phase for the work, we considered a broad spectrum of epistemic UQ methods that are theoretically suitable for dynamical systems such as UDEs. This included UQ methods based on multistart ensemble, MCMC sampling, Variational Inference, PLs/Posteriors and the FIM. In light of aspects related to scalability and applicability (e.g. due to identifiability constraints), we decided to disregard PL/Posterior calculation as well as methods using the FIM. For a detailed discussion, we refer to appendix A. The UQ methods for this study are illustrated in figure 1, and a description is provided in the following.

#### (i) Ensemble-based uncertainty quantification

To construct an ensemble-based UQ method, we consider combining ideas from deep learning [13] and mechanistic dynamical modelling [8] to define ensemble members that are customized to UDEs. After defining a prior distribution, encoding mechanistic knowledge when possible, we sample m initial values $\Theta_{\text{init}}=\left\{\theta_{\text{init}}^{1},...\theta_{\text{init}}^{m}\right\}$ from it. Then, m UDEs are trained, each starting with one element of $\Theta_{\text{init}}$ . The resulting optima are denoted by $\Theta =\left\{{\hat{\theta }}^{1},...,{\hat{\theta }}^{m}\right\}$ . During the optimization procedure, two issues have to be solved: overfitting and non-optimal local minima. To overcome overfitting, we use early stopping (in combination with an L2 regularization of the neural network parameters). Note that the necessary train-validation split yields a second source of randomness in our ensemble implementation. Additionally, like many dynamical approaches, UDEs can face convergence issues and numerical instabilities, which results in estimates with low likelihood. To address the issue of ending the optimization at non-optimal local minima, only a subset of the resulting estimators in Θ are accepted as ensemble members. We assume that m is large enough, such that the estimated maximum-likelihood estimate over all elements in Θ , ${\hat{\theta }}_{\text{MLE}}$ , is approximately equal to the theoretical maximum-likelihood estimate $\theta_{\text{MLE}}$ . Based on a likelihood-ratio test, we evaluate whether the likelihoods of the other parameters in Θ significantly differ from ${\hat{\theta }}_{\text{MLE}}$ (a method commonly used in Systems Biology [8,16]). Hence, we keep parameter values which achieve a likelihood similar to the best estimate. The test statistic is defined as


(3.3) $$\begin{array}{lcr} & \lambda (\hat{\theta })=-2(\log(p(D|\theta_{\text{MLE}}))-\log(p(D|\hat{\theta })))\approx -2(\log(p(D|{\hat{\theta }}_{\text{MLE}}))-\log(p(D|\hat{\theta }))) & \end{array}$$

and evaluated for all $\hat{\theta }\in \Theta$ . The threshold for $\lambda (\hat{\theta })$ is given by the α -quantiles of a $\chi^{2}$ distribution with $n_{f}$ degrees of freedom, where $n_{f}=1$ can provide a lower bound on the uncertainty. The parameters which meet the criterion provide an ensemble, which can be used for parameter and prediction uncertainty analysis (see [8]).

#### (ii) MCMC-based uncertainty quantification

Bayesian models perform Bayesian inference for the parameters of a model based on equation (3.1). Usually, the posterior has no closed-form solution. Instead, one relies on approximate inference algorithms like Variational Inference or MCMC-based methods [31].

MCMC-based UQ exploits parameter samples of the posterior distribution. These samples can be obtained using MCMC sampling approaches such as (adaptive) Metropolis–Hasting, parallel tempering and Hamiltonian Monte Carlo (HMC) algorithms. HMC algorithms leverage gradient information to search the parameter space efficiently, which has proven to be useful in dynamical modelling [34] and Bayesian neural networks [31]. To mitigate the problem that plain HMC is highly sensitive to parameters of the algorithm, one can use the HMC extension No-U-Turn Sampler (NUTS) [35]. At the moment, the NUTS algorithm is state-of-the-art for Bayesian neural networks [36], showing the best performance in the context of neural ODEs [22]. Yet, even with these approaches, the sampler often struggles to explore more than one mode. Parallel tempering algorithms operate with chains on different kinetic energy levels (temperatures). While low-temperature chains explore individual modes, high-temperature chains more easily traverse through the parameter space. By swapping the states of different chains under certain circumstances, parallel tempering algorithms aim to explore multimodal distributions more efficiently [37].

#### (iii) Variational Inference-based uncertainty quantification

Variational Inference approximates the posterior distribution p(θ|D) using a parametric distribution q(θ|ψ) with distribution parameters $\psi \in {\mathbb{R}}^{n_{\psi }}$ [23]. The Kullback–Leibler divergence is commonly used as an objective function, describing the discrepancy between the two distributions. This reduces the inference problem to defining an appropriate variational distribution q(θ|ψ) and estimating its parameters ψ . Commonly used options are, for example, a Gaussian distribution q(θ|ψ)=N(θ|μ,Σ) , if no parameter bounds are given, or a scaled beta distribution q(θ|ψ)=c⋅Beta(θ|a,b) if θ∈(0,c) .

## 4. Performance evaluation of methods: insights from synthetic examples

To assess the performance of the aforementioned UQ methods, we performed experiments on several synthetic problems. By using synthetic problems, we can evaluate the methods’ performance by comparing their results to the known data-generating process.

### (a) Model formulation

In this study, we considered three different models (SEIR Pulse, SEIR Waves and Quadratic Dynamics) with two different noise distributions (Gaussian and negative Binomial). For each noise distribution, we investigated several noise parameter settings. Electronic supplementary material, table S1 and appendix C provide an overview of the considered models and scenarios.

In the main part of the paper, we present the results based on the SEIR model, a compartmental model describing the dynamics of infectious diseases. This model describes the number of susceptible (S), exposed (E), infectious (I) and recovered (R) individuals using a system of differential equations:


(4.1) $$\begin{array}{lrr} & \begin{array}{rl} \frac{dS}{dt} & =-\beta (t)\frac{SI}{N},\;\;\frac{dE}{dt}=\beta (t)\frac{SI}{N}-\alpha E,\;\;\frac{dI}{dt}=\alpha E-\gamma I,\;\;\;\frac{dR}{dt}=\gamma I, \end{array} & \end{array}$$

where β is the transmission rate, α the transition rate, γ the recovery rate and N=S+E+I+R the population size.

We create synthetic data for two scenarios, differing the time-dependent transmission rate β(t) (see electronic supplementary material, figure S1):

(1) In the **SEIR Pulse** scenario, we define the transmission rate as


(4.2) $$\begin{array}{r} \beta (t)=\left\{ \begin{array}{ll} 0.05 & if\;15<t<30,\\ 0.5 & else, \end{array}\right. \end{array}$$

which emulates a time-restricted political intervention.

(2) In the **SEIR Waves** scenario, we define the transmission rate as


(4.3) $$\begin{array}{lcr} & \beta (t)=0.3\cdot \cos\left((-1+\sqrt{1+4t})\cdot 1.5+0.25\cdot \pi \right)+0.4, & \end{array}$$

which is an oscillating function for emulating the potentially complex time-dependence of contact behaviour in combination with potential virus evolution.

For the synthetic data generation, we assumed that the state variables I and R are observed at 30 time points. The parameters and initial conditions used for simulation are provided in appendix B.

In practice, the basic transmission process of an infectious disease is often known, but the parameter values and the time-dependence of the transition rate are unknown. To capture this case, we assumed the mechanistic formulation of the differential equation to be known, but the constant rate parameters α and γ as well as the time-dependent transmission rate β to be unknown. A neural network was used to model β , where we use a tanh-parameterization to ensure bounds and easily capture fast-changing dynamics. For the estimation of the noise parameter, a log transformation was used; and for α and γ , a tanh transformation was used (see appendix D).

### (b) Results

In the following, we present the results for ensemble-, MCMC- and Variational Inference-based UQ. Implementation details such as the prior definition and hyperparameter settings are provided in appendix D.

#### (i) Ensemble-based uncertainty quantification

**Figure 2.**
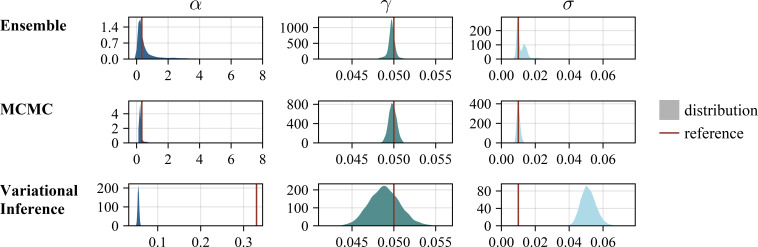
Comparison of the UQ methods on the SEIR Waves problem (Gaussian noise, σ=0.01 ), visualization of estimated parameter values. The visualization for the time-dependent parameter β(t) is provided in appendix I.

**Figure 3.**
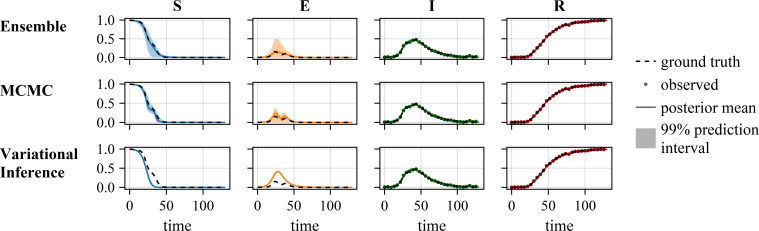
Comparison of the UQ methods on the SEIR Waves problem (Gaussian noise, σ=0.01 ), visualization of estimated trajectories.

The assessment of the results for ensemble-based UQ revealed overall a good performance: The true parameters are contained in the uncertainty intervals (figure 2) and simulated trajectories of the dynamics of the model using the true parameter values are contained in the prediction intervals (figure 3). Indeed, also the (unknown) standard deviation used for synthetic data generation are contained in the corresponding uncertainty intervals (figure 2). Yet, the long tail of the distribution indicates that the ensemble contains samples that overestimate the aleatoric uncertainty. Furthermore, while the underlying dynamics of the unobserved states could be recovered, the UQ for the transmission rate β yields broad prediction intervals (electronic supplementary material, figure S12). A broad band of trajectories of β yields reasonable values for the observed states I and R . This is not unexpected since β influences the state change only for time points with I⋅S≫0 . We would expect a reliable reconstruction only in these regimes. Ensemble members with smaller negative log-likelihood values tend to show dynamics more closely related to the dynamics of the data generation process for these time points.

We observe that, as expected, the ensemble-based UQ method yields larger prediction uncertainty bounds with increasing aleatoric noise (electronic supplementary material, figure S13). Small fluctuations in the trajectory cannot be captured easily within a setting of negative binomial noise, as is indicated by the ensemble mean trajectory for I . In the SEIR problem scenarios, the role of the neural network is well isolated from the other dynamical components. In the quadratics dynamics scenario, this is different, because the neural network can in principle completely replace known mechanistic dynamics and describe all the dynamics. Hence, the mechanistic part is only a soft constraint on the form of the whole dynamics. A consequence of this is visualized in electronic supplementary material, figure S14: the predictions of some ensemble members quickly deviate from the reference dynamics outside the data domain.

One difficulty of the ensemble-based UQ method was the choice of a reasonable threshold. As visualized in electronic supplementary material, figure S11, subselecting a fraction of the best- performing models (which is equivalent to a different significance level for the $\chi^{2}$ -test) can result in widely different confidence bands. An exemplary waterfall plot displaying the selection of the ensemble members based on likelihood values is provided in electronic supplementary material, figure S9 and shows that there is no clear convergence to a minimum objective function value for UDEs. A major advantage of the ensemble-based UQ method is its flexible parallelisability: Every candidate member of the ensemble can be trained independently of one another. For 10 000 candidate ensemble members, the training took between 4−12 h using 20 CPU cores.

#### (ii) MCMC-based uncertainty quantification

We implemented Bayesian UDEs using a NUTS and parallel tempering sampling algorithm to compare different and potentially suitable algorithms for UDEs. When sampling, the biggest issue of overparameterized models is the exploration of multiple modes. Neural networks specifically tend to have by construction various symmetries in the loss landscape [38], resulting in the possibility that no additional predictive information is added even if multiple modes are explored. We systematically experimented with different numbers of chains and samples. However, similar to what is observed with neural networks in classical supervised learning tasks [36], MCMC chains neither mix well nor properly converge in the context of UDEs. Our analysis of the sample distributions indicated that parallel tempering algorithms achieved a better exploration than NUTS. Furthermore, the initialization of the sampling at optimization endpoints—a common recommendation for dynamical systems [39]—was beneficial for UDEs.

MCMC-based UQ using NUTS and parallel tempering was computationally much more demanding than ensemble-based UQ. The generation of approximately 10 000 samples using parallel tempering required 5−7 days on a CPU node with 20 CPU cores. Yet, the resulting Bayesian confidence intervals possess a clear interpretation and there are no additional hyperparameters. Indeed, while challenging to apply, the parallel tempering runs provided posterior samples, which covered the true parameter values (figure 2). Furthermore, the corresponding prediction intervals contained the true dynamics of the process (figure 3).

#### (iii) Variational Inference-based uncertainty quantification

We performed Variational Inference using a mean-field approximation of the posterior with multivariate normal base distributions. This is a common choice for a broad range of uncertainty analysis problems [10]. Yet, the choice of approximation and base distribution can have a substantial impact on the approximation quality.

Variational Inference-based UQ required < 24 h on a single CPU core and was therefore computationally less demanding than ensemble- and MCMC-based UQ. However, the assessment of the results revealed that Variation Inference provided biased estimates. The approximated posterior distribution did not cover the true values of α and σ (figure 2). Furthermore, while the observations are fitted, Variational Inference underestimates the uncertainty on the trajectories of the unobserved state variables (figure 3).

#### (iv) Comparison of methods

The three considered methods tackle the problem of UQ from different angles. This results in differences concerning data usage and incorporation of prior knowledge, as well as posterior coverage. Since the ensemble-based UQ method is based on the optimization of an over-parametrized model, a train-validation split is necessary to implement early stopping and avoid overfitting on the training data. The combined dataset is only used for subselecting from ensemble candidate models. For MCMC- and Variational Inference-based methods, the whole dataset is used in every step of the algorithms. While the incorporation of prior knowledge in the dynamic equations, noise model and observable mapping is independent of the UQ method, assumptions about parameter values are treated differently. For the ensemble-based method, the parameter prior influences the start points of the optimization process. Afterwards, we only encode upper and lower bounds that restrict the parameter update steps. For both MCMC- and Variational Inference-based UQ, the prior distributions influence the posterior in every update step of the algorithms.

Our comparison of UQ results across methods revealed that ensemble- and MCMC-based methods provide assessments/predictions, while the standard Variational Inference method provides biased estimates and is unable to capture the dynamics of the unobserved state variables. Interestingly, while one might expect that this changes for small noise levels, our findings suggest that it holds independent of measurement noise (appendix F), which might be due to the over-parametrization in the UDE and corresponding non-identifiabilities.

**Figure 4.**
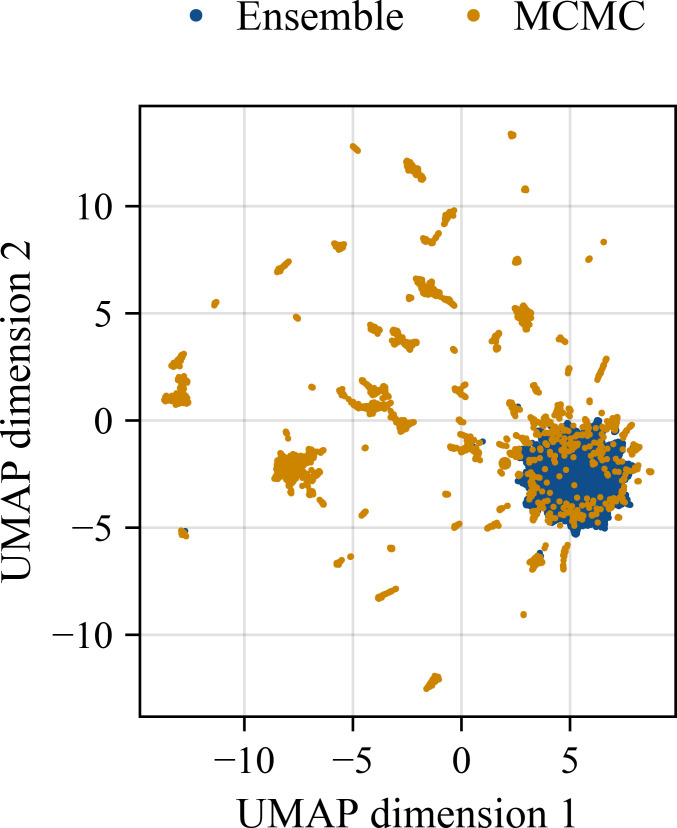
UMAP for the set of parameters obtained by the MCMC and ensemble-based method. The parameter vectors contained mechanistic and neural network parameters.

**Figure 5.**
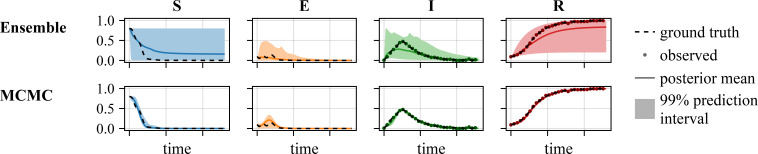
Comparison of prediction performance using the same setting as in figure 3 but with a new initial condition $x_{0}=\left(0.8,0.1,0.0,0.1\right)$ .

Yet, we also identified substantial differences between ensemble- and MCMC-based UQ method results. A dimension reduction of the parameter samples shows different characteristics. The uniform manifold approximation and projection (UMAP) [40] of the collection of all samples using parameter values as features reveals that: (i) ensembles derived from optimization results cluster; and (ii) parallel tempering yields a more patchy pattern and several additional clusters (figure 4). While on first glance this suggests that parallel tempering achieves a better exploration, it also seems to miss high-quality parameter vectors identified using optimization. To assess this further, we studied the model predictions for an initial condition for which no measurements were available (figure 5). The ensemble-based UQ yields substantially broader intervals than the MCMC-based UQ. Predictions of the MCMC-based UQ still cover the model simulation for the true data.

## 5. Conclusions and future perspectives

UDEs model the dynamics of observed and unobserved states using a combination of interpretable mechanistic and ANN parameters. UQ for both mechanistic parameters and state trajectories is critically important, especially in fields like healthcare and epidemiology. However, performing UQ for UDEs poses unique challenges, primarily due to the high dimensionality of the parameter space and the complex structure of the loss or posterior function landscape.

Our assessment of UQ methods revealed that ensemble- and MCMC-based methods perform better than Variational Inference. This observation holds true across different noise levels. Nevertheless, defining a suitable threshold for ensemble-based methods requires further investigation, and the convergence of MCMC methods remains problematic.

Our findings underscore the need for further methodological developments. Building upon the work of [36], developing methods for automatic symmetry removal could improve computational efficiency in UQ for UDEs and enable scaling to larger problems. Furthermore, exploring hybrid UQ approaches that combine the resource efficiency of ensemble methods with the statistical interpretability of MCMC-based methods might be advantageous. Lastly, investigating model structure uncertainty may provide valuable insights into how mechanistic terms are absorbed by the neural network.

The results of our study likely extend beyond UDEs and may also apply to alternative machine learning-based modelling approaches. An assessment of ANNs designed to control dynamical systems [41] would be of particular interest. Furthermore, extending this work to stochastic models, particularly neural stochastic differential equations [42], would be beneficial. Accordingly, while this study provides novel insights, it can only serve as a starting point for further research.

## Data Availability

Code to run the presented experiments and regenerate simulation data can be found in the Github repository [43]. Supplementary material is available online [44].
